# The RING-Type Domain-Containing Protein GNL44 Is Essential for Grain Size and Quality in Rice (*Oryza sativa* L.)

**DOI:** 10.3390/ijms25010589

**Published:** 2024-01-02

**Authors:** Lei He, Tao Chen, Wenhua Liang, Chunfang Zhao, Ling Zhao, Shu Yao, Lihui Zhou, Zhen Zhu, Qingyong Zhao, Kai Lu, Cailin Wang, Li Zhu, Yadong Zhang

**Affiliations:** 1 Institute of Food Crops, Key Laboratory of Jiangsu Province for Agrobiology, East China Branch of National Center of Technology Innovation for Saline-Alkali Tolerant Rice, Jiangsu Academy of Agricultural Science, Nanjing 210014, Chinaclwang@jaas.ac.cn (C.W.); 2 Zhongshan Biological Breeding Laboratory, Nanjing 210014, China; 3 State Key Laboratory of Rice Biology, China National Rice Research Institute, Hangzhou 311400, China

**Keywords:** appearance quality, eating and cooking quality, grain yield, grain width, rice

## Abstract

Grain size in rice (*Oryza sativa* L.) shapes yield and quality, but the underlying molecular mechanism is not fully understood. We functionally characterized *GRAIN NUMBER AND LARGE GRAIN SIZE 44* (*GNL44*), encoding a RING-type protein that localizes to the cytoplasm. The *gnl44* mutant has fewer but enlarged grains compared to the wild type. *GNL44* is mainly expressed in panicles and developing grains. Grain chalkiness was higher in the *gnl44* mutant than in the wild type, short-chain amylopectin content was lower, middle-chain amylopectin content was higher, and appearance quality was worse. The amylose content and gel consistency of *gnl44* were lower, and protein content was higher compared to the wild type. Rapid Visco Analyzer results showed that the texture of cooked *gnl44* rice changed, and that the taste value of *gnl44* was lower, making the eating and cooking quality of *gnl44* worse than that of the wild type. We used *gnl44*, *qgl3*, and *gs3* monogenic and two-gene near-isogenic lines to study the effects of different combinations of genes affecting grain size on rice quality-related traits. Our results revealed additive effects for these three genes on grain quality. These findings enrich the genetic resources available for rice breeders.

## 1. Introduction

Rice (*Oryza sativa* L.) is an important food crop that provides the daily dietary calories for much of the human population; as such, its yield is crucial to ensuring food security. Grain weight, grain number per panicle, and panicle number per plant are the most important agronomic components of grain yield. Numerous genes regulating grain number have been identified in rice over the years [1,2,3,4,5]. These genes function in various periods of spike development. Nevertheless, knowledge of the genetic and molecular mechanisms that establish grain number in rice is still limited.

Grain weight is determined by the combination of grain length, width, and thickness. Many quantitative trait loci (QTLs) and genes related to grain size have been functionally characterized in rice. Grain size-related genes that have been cloned to date affect one of five pathways: the ubiquitin proteasome pathway, phytohormone signaling or biosynthesis, transcriptional regulation, mitogen-activated protein kinase (MAPK) signaling, or G protein signaling [6]. For instance, *GRAIN WIDTH 2* (*GW2*) encodes a RING-type protein that negatively regulates grain width, as plants carrying a loss-of-function *gw2* allele produce wider grains [7]; *DECREASED GRAIN SIZE1* (*DGS1*) encodes a C_3_HC_4_-type RING protein that regulates rice grain size and participates in the ubiquitin proteasome pathway [8]. *PROTEIN PHOSPHATASE WITH KELCH-LIKE REPEATS 1* (*OsPPKL1*), also named *GRAIN LENGTH 3.1* (*GL3.1*), encodes a protein phosphatase containing two Kelch domains that negatively regulate rice grain length through a modulation of brassinosteroid (BR) signaling [9]. *GW5* and *GRAIN SIZE 5* (*GS5*), *OsGSK5*/*TGW3*/*GL3.3*, and *OsGSK2* are also involved in BR signaling [10,11,12,13]. Similarly, *THOUSAND GRAIN WEIGHT 6* (*TGW6*) and *BIG GRAIN 1* (*BG1*) regulate grain size through auxin signaling [14,15]. *Grain Length, Width and Weight 7* (*GLW7*) encodes the plant-specific transcription factor OsSPL13, which regulates grain size by controlling cell proliferation in the spikelet hull [16]. The MAPK kinase kinase 10–MAPK kinase 4–MAPK6 (OsMKKK10–OsMKK4–OsMAPK6) regulatory module plays an important role in regulating grain size [4,17,18]. GRAIN SIZE AND NUMBER 1 (GSN1), also named MAPK PHOSPHATASE 1 (OsMKP1), negatively regulates the OsMKKK10–OsMKK4–OsMPK6 cascade to coordinate the balance between grain size and grain number. The rice genome harbors one gene each for *Gα* and G*β* and five *Gγ* genes. Both Gα and Gβ proteins can positively regulate grain size [19]. Gγs can be classified into three distinct types according to their C-terminal structures. Group I (RGG1) and Group II (RGG2) participate in the regulation of abiotic stress [20,21]. DENSE AND ERECT PANICLE 1 (DEP1), GGC2, and GRAIN SIZE3 (GS3) belong to Group III and can regulate grain size and rice yield. Moreover, the manipulation of *DEP1*, *GGC2*, or *GS3* transcript levels can affect grain size [22,23,24].

In addition to yield, grain size can also affect the quality of rice. In general, large grains are associated with high yield but poor quality, especially the appearance quality of milled rice (grains with the outer hull removed), which includes chalkiness [25,26]. Slender rice is popular with consumers for its transparent appearance and lack of chalkiness. The genotype at *GW7* can improve rice quality by changing the pattern of cell division, making rice grains more slender and less chalky ^18^. Although several grain size genes have been cloned in rice, the genetic and molecular mechanisms that determine grain size remain largely unknown [27]. Functional identification of other grain size genes or QTLs is important to further our understanding of the molecular mechanisms regulating grain-related traits and to help meet the demand for high-yielding and superior-quality cultivars.

Although numerous QTLs/genes related to grain size have been functionally characterized, there have been few studies about the genetic interactions of these grain size-related genes [28]. Gao et al. (2015) used the near-isogenic lines (NILs) NIL-*GS3*, NIL-*qgl3*, and NIL-*GS3*/*qgl3* to study the interaction effects of *GS3* and *qGL3*, finding that *GS3* and *qGL3* have an additive effect on regulating rice grain length [29]. He et al. (2023) used NILs that differed by a single gene or two genes to study the genetic effects of the grain size-related genes *gw2*, *gs3*, and *qgl3* on grain size, finding that combinations of these three genes had additive effects on grain size [30]. These studies focused on the effects of grain size-related genes on grain size and yield. However, less is known about the effects of grain size-related genes on rice quality.

Here, we report that *GNL44*, encoding a RING-type E3 ubiquitin ligase, regulates grain size and quality in rice. We analyzed the effects of *gnl44*, *qgl3*, and *gs3* on rice quality-related traits using monogenic and two-gene NILs. Our results revealed additive effects for these three genes on grain quality. The findings may facilitate our understanding of the mechanisms underlying the determination of grain size and quality. Our results also provide the necessary theoretical basis and genetic resources for breeding high-yield and superior-quality rice cultivars.

## 2. Results

### 2.1. The gnl44 Mutant Produces Fewer but Larger Grains

**Figure 1:**
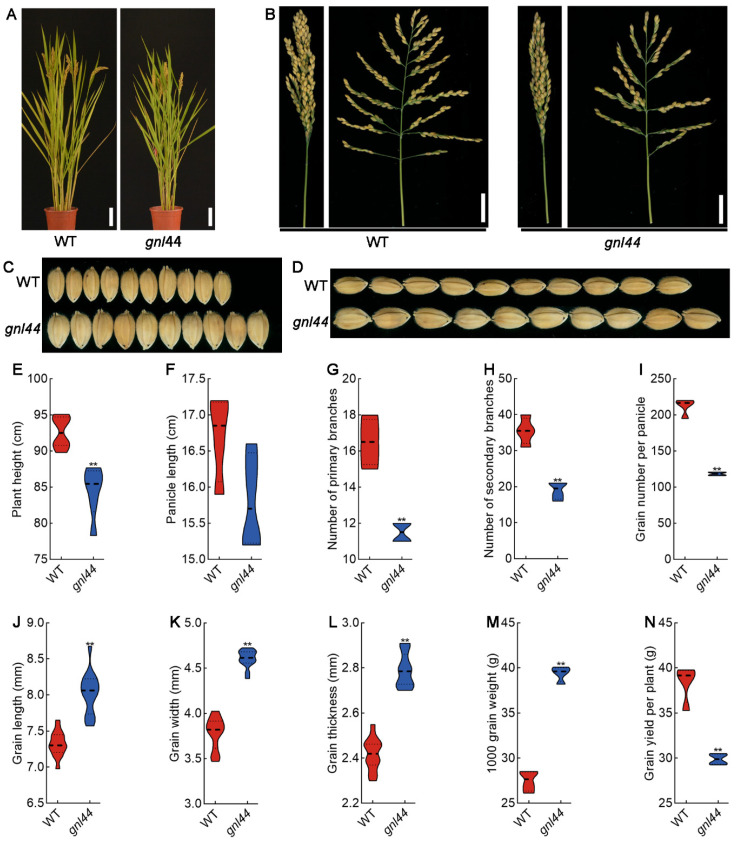
Phenotypes of WT and *gnl44* rice plants. (**A**) Morphology of WT and *gnl44* plants. Scale bars: 10 cm. (**B**) Panicles of the WT and *gnl44*. Scale bars: 3 cm. (**C**,**D**) Morphology of mature grains from the WT and *gnl44*. (**E**–**I**) Plant height (**E**), panicle length (**F**), number of primary branches (**G**), number of secondary branches (**H**), and grain number per panicle (**I**) (*n* = 10). (**J**–**N**) Grain length (**J**), grain width (**K**), grain thickness (**L**), and thousand-grain weight (**M**) of mature grains and grain yield per plant (**N**) from the WT and *gnl44* (*n* = 10). Values are means ± SD; Student’s *t*-test was used to calculate the *p* values; ** *p* < 0.01.

The *gnl44* mutant was isolated from EMS-mutagenized M_2_ populations of the WT *japonica* cultivar Wuyunjing. *gnl44* mutant plants were slightly shorter than the WT (Figure 1A,E). Panicle length, the number of primary branches, and the number of secondary branches were also lower in *gnl44* compared to the WT (Figure 1B,F–H). The grain number per panicle of *gnl44* was about 43.95% lower than that of the WT (Figure 1I). In addition, the *gnl44* mutant produced large grains (Figure 1C,D). The length of *gnl44* grains increased by 9.8% (Figure 1J) and their width increased by 21.2% compared to the WT (Figure 1K). The thickness of the *gnl44* grains increased by 15.3% compared to the WT (Figure 1L). The *gnl44* grains were also significantly heavier than the WT grains (Figure 1M). However, the grain yield per plant in *gnl44* was decreased by about 21.8% compared to the WT (Figure 1N). These results indicate that *gnl44* negatively regulates grain number and grain size in rice.

### 2.2. GNL44 Affects Cell Division and Cell Expansion

**Figure 2:**
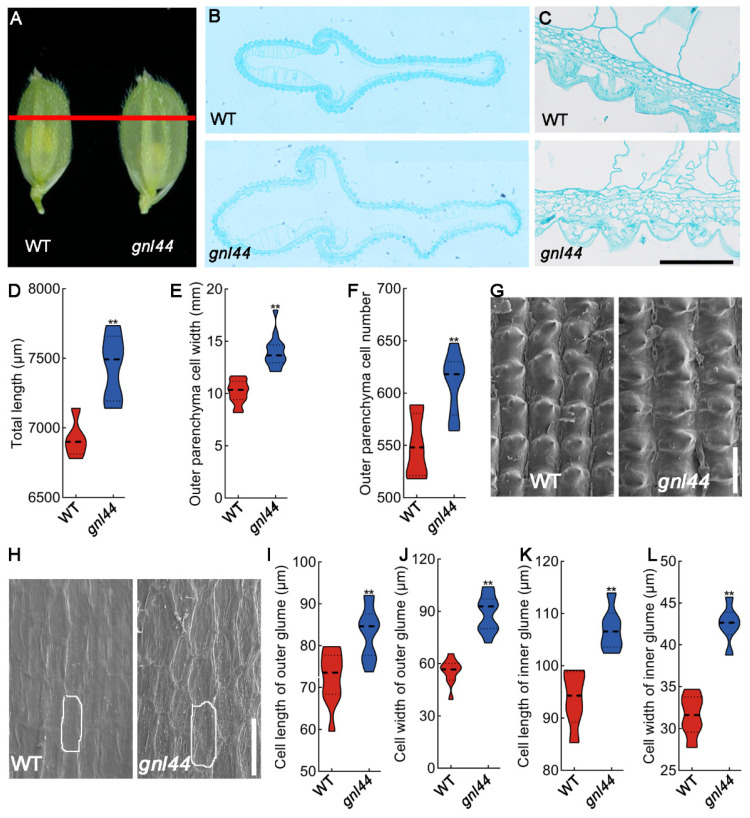
Histological analysis of spikelet hulls in the WT and *gnl44.* (**A**) Young spikelet hulls of the WT and *gnl44*. The red line indicates the position of the cross-section shown in (**B**). (**B**) Cross-sections of spikelet hulls (10×). (**C**) Magnified view of the cross-section in ((**B**), 40×); scale bar: 50 μm. (**D**–**F**) Total length (**D**), cell width (**E**), and cell number (**F**) in the outer parenchyma layer (*n* = 20). (**G**,**H**) Scanning electron microscopy analysis of the outer ((**G**), 120×) and inner ((**H**), 150×) surfaces of glumes. Scale bars: 100 μm. (**I**–**L**) Cell length (**I**,**K**) and width (**J**,**L**) in outer (**I**,**J**) and inner (**K**,**L**) glumes (*n* = 50). Values are means ± SD; Student’s *t*-test was used to calculate the *p* values; ** *p* < 0.01.

Given that *gnl44* had longer and wider grains surrounded by the spikelet hull compared to the WT (Figure 2A), we produced paraffin sections from developing grains to analyze the parenchyma cell layer in *gnl44* and WT. The length of the outer parenchyma cell layer and the number of cells were significantly increased in *gnl44* compared to the WT (Figure 2B–E). In addition, we measured the length and width of cells in the outer and inner glumes of WT and *gnl44*, revealing that the outer epidermal cells in *the gnl44* lemmas were longer and wider than those of the WT. Similarly, the inner epidermal cells of *gnl44* were longer and wider than those of the WT (Figure 2F–K). Thus, GNL44 regulates grain size by limiting cell expansion in spikelet hulls.

### 2.3. Cloning of GNL44

To determine the genetic basis of the *gnl44* phenotype, we crossed the *gnl44* mutant with the WT rice cultivar WYJ. All F_1_ plants showed a wild-type phenotype (normal grains), and the segregation (normal grains: large grains) ratio in the F_2_ progeny was 3:1 (Table 1). These results suggest that the *gnl44* mutation is recessive and affects a single nuclear gene.

**Figure 3:**
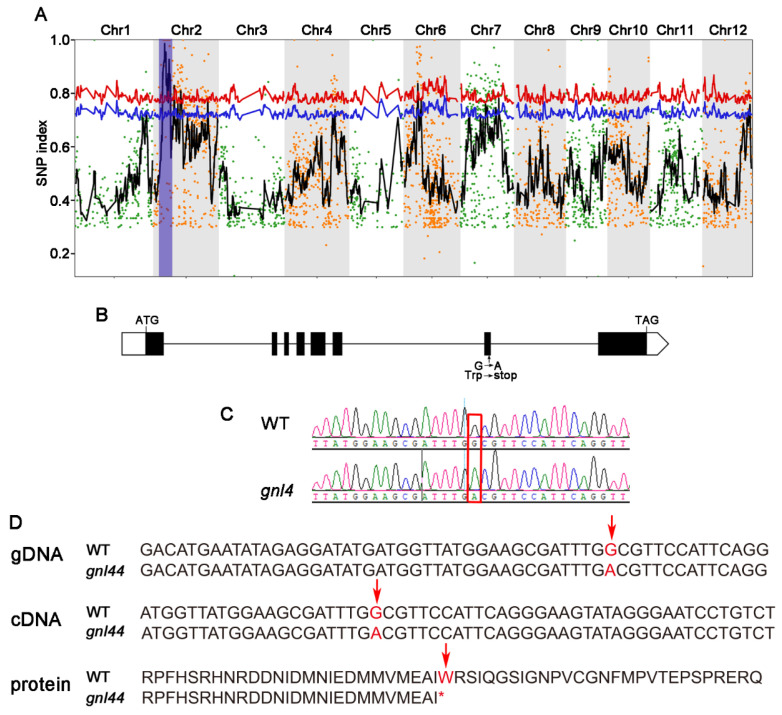
Positional cloning of *GNL44.* (**A**) SNP index and corresponding two-sided confidence intervals: 95% (blue line) and 99% (red lines). Different color dots are the original values of the SNP index of different chromosomes, and the black curve are the fitted values of SNP index. The regions indicated in blue shading correspond to the candidate genomic interval. (**B**) *GNL44* gene structure. Black boxes: exons; white boxes: untranslated regions; black lines: introns. ATG and TGA represent the start codon and the stop codon, respectively. (**C**) Sanger sequencing electropherograms for the WT and *gnl44*. The mutation site is highlighted by the red box. (**D**) Mutation in *GNL44* genomic DNA, the *GNL44* cDNA, and in the predicted GNL44 or gnl44 protein in the WT and *gnl44*, arrow indicated the mutation site, * indicated the stop codon.

We cloned *GNL44* using the MutMap approach. We sequenced genomic DNA from a pool of F_2_ plants with the *gnl44* phenotype from the *gnl44* × WYJ cross. We quantified the frequency of single-nucleotide polymorphisms (SNPs) in the sequencing results, as described previously [2]. We detected 2979 SNPs and 227 small insertions and deletions (InDels) between WYJ and the pooled F_2_ plants with the *gnl44* phenotype. Using these SNPs, we calculated the SNP index in the pooled F_2_ plants across the entire genome (Figure 3A). The mapping interval of *GNL44* on chromosome 2 is 520 kb. Of all SNPs in this interval, only one located in the coding region of a predicted gene had an SNP index of 1, with clear and tight linkage with neighboring SNPs. This SNP in *gnl44* is a mutation from A to G located in the 7th exon of *Os02g0244100*, causing a premature termination of translation (Figure 3B–D). These results indicate that *Os02g0244100* is the candidate gene for *GNL44*.

### 2.4. Complementation Test

To test whether the mutation in *Os02g0244100* was responsible for the observed *gnl44* phenotypes, we carried out a genetic complementation test. We introduced a WT genomic fragment containing the entire coding region of *GNL44* together with 1662 bp of upstream and 787 bp of downstream sequence into the *gnl44* mutant by Agrobacterium-mediated transformation, yielding 5 transgenic lines (GNL44COM/*gnl44*).

**Figure 4:**
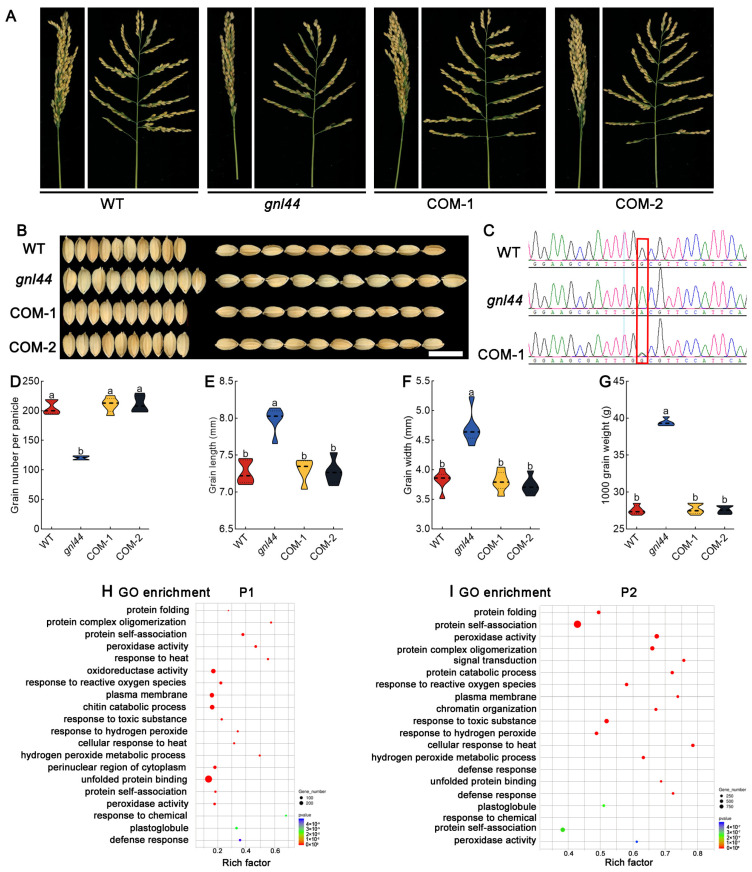
Complementation of the *gnl44* mutant. (**A**) Photographs of panicle from the WT, *gnl44*, and two independent *GNL44* complementation lines (COM-1, COM-2). (**B**) Morphology of mature grains from the WT, the *gnl44* mutant, and the two complementation lines. Scale bar: 1 cm. (**C**) Sanger sequencing electropherograms for the WT, *gnl44,* and COM-1. The mutation site is highlighted by the red box. (**D**–**G**) Grain number per panicle (**D**), grain length (**E**), grain width (**F**), and thousand-grain weight (**G**) of mature grains of A (*n* = 10). Values are means ± SD. Different letters indicate significant differences as determined by analysis of variance (ANOVA) followed by an LSD test for the comparison of means (*p* < 0.05). (**H**,**I**) Gene Ontology (GO) enrichment analysis of differentially expressed genes (DEGs) between WT and *gnl44* at different stages of panicle development.

We quantified similar values for grain number per panicle, grain length, grain width, and thousand-grain weight in the WT and the complementation lines (Figure 4A–G). These results indicate that *GNL44* is *Os02g0244100*, also named *GW2*. *GNL44* is annotated by The Rice Annotation Project (rapdb.dna.affrc.go.jp, accessed on 6 May 2021) as encoding a RING-type E3 ubiquitin ligase.

To study the molecular mechanism underlying the role of *GNL44* in grain-type formation, we performed transcriptome sequencing of panicles at different stages of development. We prepared and sequenced RNA samples from WT and *gnl44* panicles at stages P1 (3 cm) and P2 (10 cm). The results indicated that the target genes of *GNL44* are mainly involved in protein metabolism pathways such as protein folding, protein self-association, and protein complex oligomerization (Figure 4H,I). Peroxidase activity and oxidoreductase activity were also significantly enriched (Figure 4H,I). These results suggest that the *GNL44* mutation has a significant impact on important biological processes such as protein-related metabolic pathways and redox pathways, thereby affecting the growth and development of glumes during the early stage of panicle development.

### 2.5. Expression of GNL44 and Subcellular Localization of GNL44

**Figure 5:**
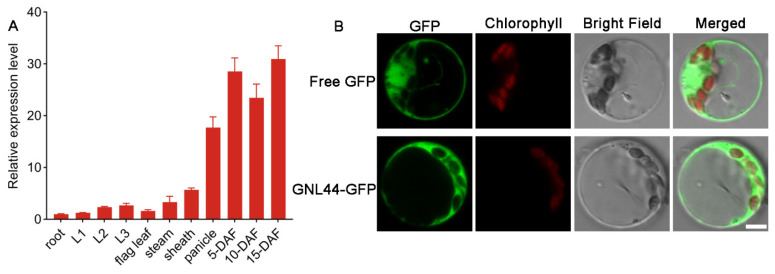
*GNL44* expression pattern in the WT and subcellular localization of GNL44-GFP. (**A**) Relative *GNL44* transcript levels in various organs of WT plants. L1–L3: first–third leaf of rice seedlings; DAF: days after fertilization. The expression in roots was set to 1. (**B**) Subcellular localization of GNL44-GFP in transfected rice protoplasts. Free GFP from the p35S:GFP construct served as control. GFP: GFP fluorescence; chlorophyll: chlorophyll autofluorescence. Scale bar: 10 μm.

We measured *GNL44* expression in different tissues collected from WT plants and observed that *GNL44* was preferentially expressed in panicles and developing seeds (Figure 5A). To assess the subcellular localization of GNL44, we transfected rice protoplasts with a construct encoding a GNL44–green fluorescent protein (GFP) fusion or with *35S:GFP* as control (Figure 5B). We detected green fluorescence for GNL44-GFP in the cytoplasm, indicating that GNL44 is a cytosolic protein.

### 2.6. GNL44 Affects Grain Appearance Quality of Milled Rice

**Figure 6:**
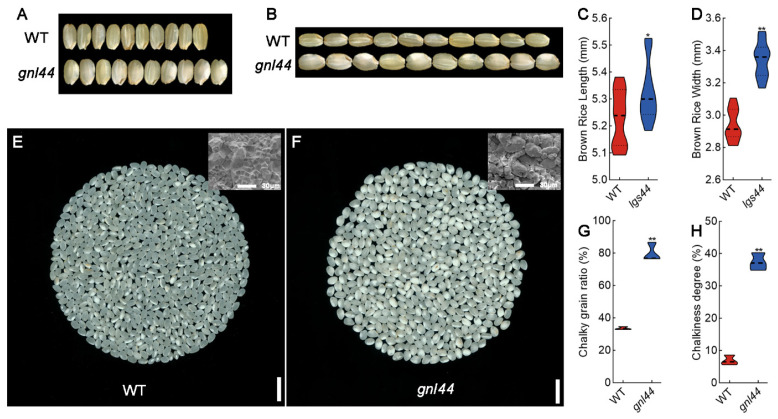
Grain appearance quality of WT and *gnl44* milled rice. (**A**,**B**) Photographs showing the length (**A**) and width (**B**) of brown rice from the WT and *gnl44*. Scale bars: 10 mm. (**C**,**D**) Length (**C**) and width (**D**) of brown rice from the WT and *gnl44*. Values are means ± SD (*n*  =  3). (**E**,**F**) Increased chalkiness in milled rice of *gnl44* compared to the WT. Scale bars: 10 mm. Scanning electron microscopy of cross-sections of milled rice from the WT and *gnl44* are shown in upper right corners (scale bars, 30 μm). (**G**,**H**) Percentage of milled rice with chalkiness (**G**) and chalkiness degree of milled rice from the WT and *gnl44* (**H**). Student’s *t*-test was used to calculate the *p* values; ** *p* < 0.01, * *p* < 0.05.

To explore the effect of the *GNL44* genotype on rice quality, we characterized the appearance of rice from WT and *gnl44*. The width and length of brown grains from *gnl44* were higher than those from the WT (Figure 6A–D). The transparency of the milled rice from *gnl44* was lower than that from WT. Some milled rice from *gnl44* showed large areas with a white core in endosperm (Figure 6E,F). In agreement, chalky grain rate and chalkiness degree in the *gnl44* mutant were significantly higher than in WT (Figure 6G,H). To gain a better picture of grain ultrastructure, we observed the structure of starch granules from WT and *gnl44* grains by scanning electron microscopy. The starch granules of WT were closely arranged and regularly organized in contrast to the starch granules of *gnl44*, which were irregular and loosely arranged (Figure 6E,F). These results suggest that the appearance quality of milled rice is reduced in the *gnl44* mutant.

### 2.7. Eating and Cooking Quality of the gnl44 Mutant Is Worse Than That of the WT

**Figure 7:**
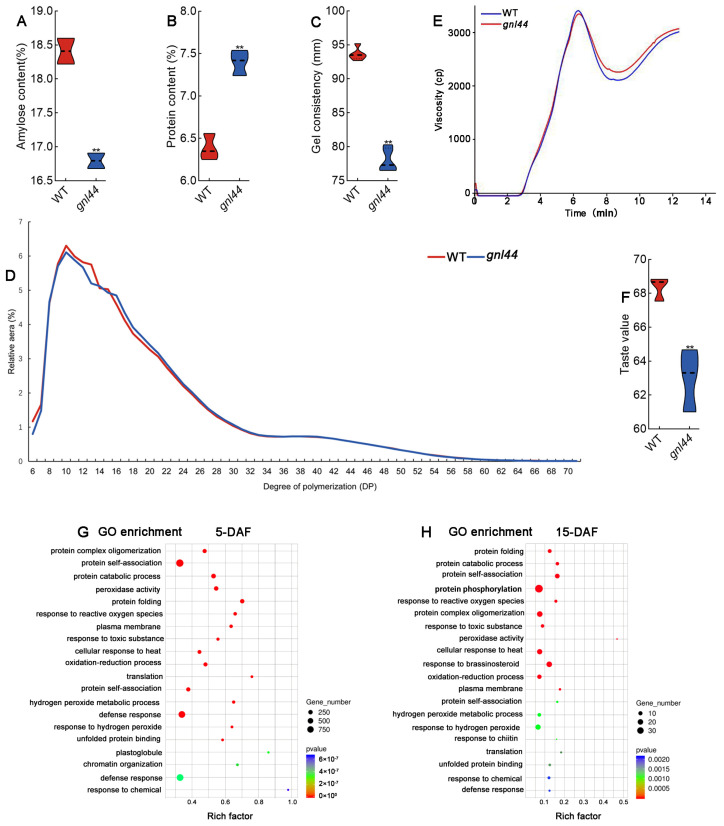
ECQ of the WT and *gnl44.* (**A**–**C**) Amylose content, protein content, and gel consistency from WT and *gnl44* flour. (**D**) Chain length distribution of amylopectin in the WT and *gnl44*. (**E**) RVA spectra of WT and *gnl44* flour. (**E**) Rapid Visco Analyzer profile characteristics of WT and *gnl44*. (**F**) Taste value of WT and *gnl44* grains. Values are means ± SD (*n* = 3 in (**A**–**C**,**F**,**G**)). Student’s *t*-test was used to calculate the *p* values; ** *p* < 0.01. (**G**,**H**) Gene Ontology (GO) enrichment analysis of differentially expressed genes (DEGs) between WT and *gnl44* at different stages after flowering.

To clarify the effects of *GNL44* on eating and cooking quality, we performed a rice quality assay. Rice flour produced from *gnl44* grains had lower amylose content, higher protein content, and lower gel consistency compared to the WT (Figure 7A–C).

The length distribution of amylopectin chains is important for determining the physicochemical properties of starch as well as the texture characteristics of cooked rice. Amylopectin can be divided into four types based on chain length: fa chains (degree of polymerization [DP] 6–12), fb1 chains (DP 13–24), fb2 chains (DP 25–36), and fb3 chains (DP > 37) [31,32]. The hardness of cooked rice is negatively correlated with the proportion of shorter fa chains present in branched starch but positively correlated with the proportion of fb2 chains. A higher proportion of long-chain branched starch increases the hardness and reduces the stickiness of cooked rice [33,34].

We measured the length distribution of amylopectin chains in rice flour produced from WT and *gnl44* grains. WT flour had higher contents of DP 6–12 amylopectin chains than *gnl44* flour, except for DP 8 chains. By contrast, WT flour had fewer DP 13–24 chains than *gnl44* flour did, except for DP 15. The contents of DP 25–36 chains were slightly lower in the WT than in *gnl44*, except for DP 36, while aggregates of DP >37 chains accumulated to similar levels in the WT and *gnl44* (Figure 7D). These results suggest that *gnl44* mainly affects the biosynthesis of fa and fb1 chains and that the grain quality of milled rice is reduced in the *gnl44* mutant.

Amylose content, protein content, gel consistency, and length distribution of amylopectin chains are all closely related to eating and cooking quality (ECQ), which prompted us to understand the effect of *gnl44* on the texture of cooked rice by carrying out an RVA experiment. The values for peak viscosity and breakdown viscosity of *gnl44* were lower than those of the WT. However, cold viscosity, final viscosity, and setback viscosity all increased in *gnl44* relative to the WT (Figure 7E, Table S2). Furthermore, the taste value of *gnl44* was lower than that of the WT (Figure 7F). These results suggest that the ECQ of *gnl44* is decreased compared to that of the WT.

The above results indicate that the mutation in *GNL44* in rice leads to poor appearance quality and lower ECQ. To determine which genes might mediate this effect, we explored the downstream target genes of GNL44 by RNA-seq analysis. We collected seeds from WT and *gnl44* plants at 5 and 15 days after flowering (DAF) for transcriptome analysis. Protein metabolism pathways such as protein folding, protein catabolic process, and protein complex oligomerization as well as redox pathways such as peroxidase activity and response to reactive oxygen species (ROS) were enriched among the differentially expressed genes (DEGs) (Figure 7G,H). These results indicate that *GNL44* affects rice appearance quality and ECQ via protein-related metabolic pathways and redox-related biological processes.

### 2.8. Rice Quality of NIL-gnl44, NIL-gs3, and NIL-qgl3

High yield and superior quality are the primary goals of rice breeders and can be affected by grain size [35]. We previously reported that the large-grain rice cultivar TD70 (thousand-grain weight of 68.6 g) carries superior alleles at the loci *qGL3* and *GS3*, which control grain size, while Kasalath (thousand-grain weight of 19.1 g) has normal alleles at these loci. TD70 is therefore an ideal material for studying the effects of genetic interactions between different grain size genes on milled rice quality [36].

We wished to explore the effect of the interaction between *GNL44* and *qGL3* and *GS3* on rice quality by constructing monogenic NILs of *qgl3* and *gs3* with Kasalath as the receptor and TD70 as the donor. We also constructed NIL-*gnl44* with Kasalath as the receptor and the *gnl44* mutant as the donor.

**Figure 8:**
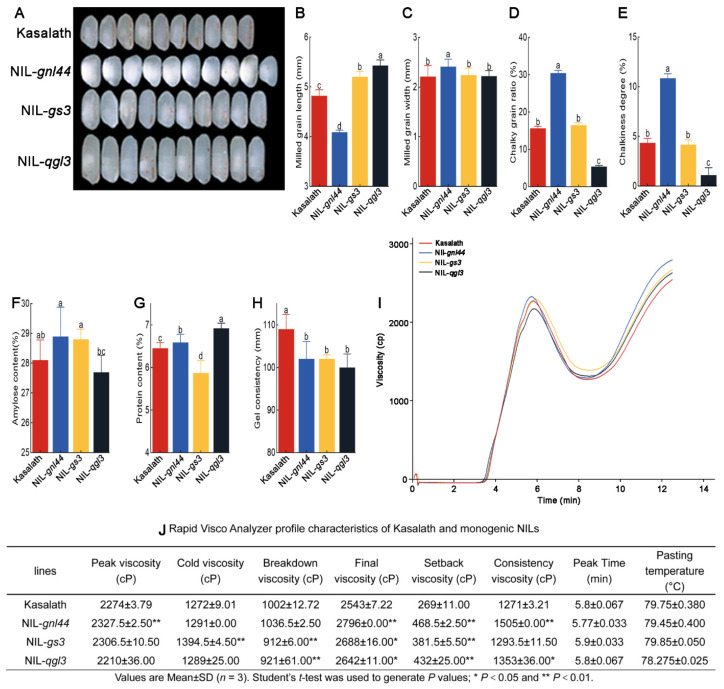
Grain quality of monogenic NILs. (**A**) Photographs showing milled rice from Kasalath, NIL-*gnl44*, NIL-*qgl3*, and NIL-*gs3*. (**B**–**H**) Milled grain length (**B**), milled grain width (**C**), chalky grain ratio (**D**), chalkiness degree (**E**), amylose content (**F**), protein content (**G**), and gel consistency (**H**) of Kasalath, NIL-*gnl44*, NIL-*qgl3*, and NIL-*gs3* grains or rice flour. Values are means ± SD (*n*  =  10 in (**B**,**C**); *n*  =  5 in (**D**,**E**); *n* = 3 in (**F**,**G**); *n* = 4 in (**H**)). (**I**) RVA spectra of Kasalath and monogenic NILs. (**J**) RVA profile characteristics of Kasalath and monogenic NILs. Different letters indicate significant differences as determined by ANOVA followed by an LSD test for the comparison of means (*p* < 0.05).

The width of the milled grain from NIL-*gnl44* and the length of the milled grain from NIL-*qgl3* were significantly higher compared to that of Kasalath (Figure 8A–C). The chalky grain ratio and chalkiness degree of NIL-*gnl44* were higher than those of Kasalath, and those of NIL-*qgl3* were significantly lower than those of Kasalath (Figure 8D,E). A rice quality assay showed that the amylose contents of NIL-*gnl44* and NIL-*gs3* were higher than that of Kasalath. The protein contents of NIL-*gnl44* and NIL-*qgl3* were also higher than that of Kasalath, while that of NIL-*gs3* was lower than that of Kasalath (Figure 8F,G). The Gel consistency of the three NILs was lower than that of Kasalath (Figure 8H). Moreover, the RVA experiments showed that the peak viscosity of NIL-*gnl44* and NIL-*gs3* were higher, and that of NIL-*qgl3* was lower, compared to that of Kasalath. The cold viscosity of NIL-*gs3* was elevated compared to that of Kasalath but was similar to that of Kasalath in NIL-*gnl44* and NIL-*gs3*. The breakdown viscosities of NIL-*gs3* and NIL-*qgl3* were lower than that of Kasalath. The final viscosity and setback viscosity were higher in all three monogenic NILs relative to Kasalath. The consistency viscosity of NIL-*gnl44* was elevated, similar in NIL-*gs3*, and slightly increased in NIL-*qgl3* compared to that of Kasalath (Figure 8I,J). These results suggest that *GNL44* and *qGL3* mainly affect the chalkiness and gel consistency and that *GS3* mainly affects protein content.

### 2.9. Additive Effects of gnl44, gs3, and qgl3 on Rice Quality

**Figure 9:**
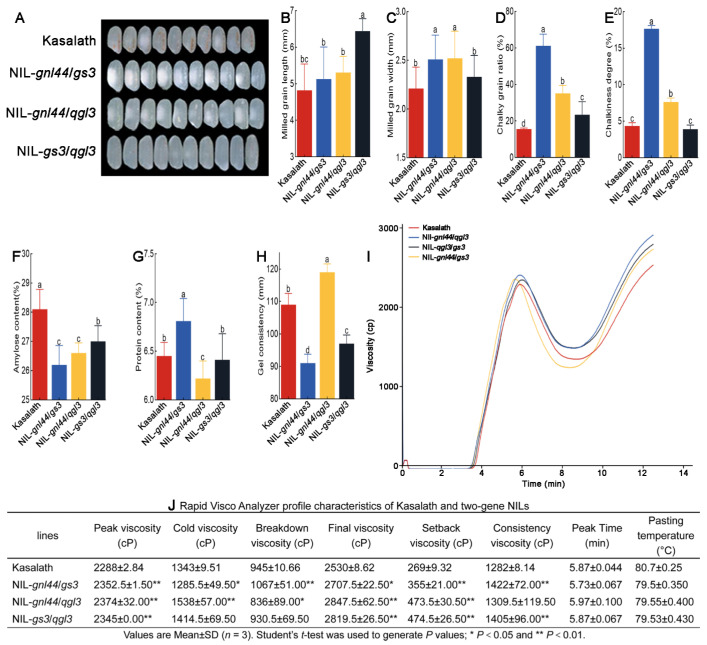
Grain quality of two-gene NILs. (**A**) Photographs showing milled rice from Kasalath, NIL-*lgs44*/*qgl3*, NIL-*lgs44*/*gs2*, and NIL-*qgl3*/*gs3*. (**B**–**H**) Milled grain length (**B**), milled grain width (**C**), chalky grain ratio (**D**), chalkiness degree (**E**), amylose content (**F**), protein content (**G**), and gel consistency (**H**) of Kasalath, NIL-*lgs44*/*qgl3*, NIL-*lgs44*/*gs2*, and NIL-*qgl3*/*gs3* grains or flour. Values are means ± SD (*n*  =  10 in (**B**,**C**); *n*  =  5 in (**D**,**E**); *n* = 3 in (**F**,**G**); *n* = 4 in (**H**)). (**I**) RVA spectra of Kasalath and two-gene NILs. (**J**) RVA profile characteristics of Kasalath and two-gene NILs. Different letters indicate significant differences as determined by ANOVA followed by an LSD test for the comparison of means (*p* < 0.05).

We also constructed two-gene NILs to evaluate the effects of different combinations of grain size genes on rice quality. The length and width of milled grain from these two-gene NILs increased in general compared to those of Kasalath (Figure 9A–C). The chalky grain ratio and chalkiness degree of the two-gene NILs NIL-*gnl44*/*gs3* and NIL-*gnl44*/*qgl3* in which *gnl44* was pyramided were higher than those of the corresponding monogenic NILs NIL-*gs3* and NIL-*qgl3*. However, the chalky grain ratio and chalkiness degree of the two-gene NILs NIL-*gnl44*/*qgl3* and NIL-*gs3*/*qgl3* in which *qgl3* was pyramided were lower than those of the corresponding monogenic NILs NIL-*gnl44* and NIL-*gs3* (Figure 9D,E). The amylose content of all three two-gene NILs was decreased compared to that of Kasalath (Figure 9F). The protein content of the two-gene NILs NIL-*gnl44*/*gs3* and NIL-*gs3*/*qgl3* was higher than that of the corresponding monogenic NILs NIL-*gnl44* and NIL-*qgl3* (Figure 9G). The gel consistency of NIL-*gnl44*/*gs3* and NIL-*gs3*/*qgl3* was lower than that of the corresponding monogenic NILs NIL-*gnl44* and NIL-*qgl3*. The gel consistency of NIL-*gnl44*/*qgl3* was the highest among the seven lines (three two-gene NILs, three monogenic NILs, and Kasalath) (Figure 9H).

RVA results showed that the peak viscosity was elevated in all two-gene NILs relative to their corresponding monogenic NILs and Kasalath. The cold viscosity of NIL-*gnl44*/*qgl3* was higher, of NIL-*gs3*/*qgl3* was similar, and of NIL-*gnl44*/*gs3* was slightly decreased relative to that of Kasalath (Figure 9I,J). The breakdown viscosity increased in NIL-*gnl44*/*gs3* and slightly decreased in NIL-*gnl44*/*gs3* compared to Kasalath. The final viscosity and setback viscosity increased for all three two-gene NILs relative to Kasalath. The consistency viscosity of NIL-*gnl44*/*gs3* and NIL-*gs3*/*qgl3* also increased (Figure 9I,J). Taken together, these findings suggest that *GNL44*, *GS3*, and *qGL3* differentially affect rice quality.

We performed a two-way analysis of variance (ANOVA) to dissect the genetic interactions between *gnl44*, *qgl3*, and *gs3* (Table 2 and Table S3). We determined that the combination of *gnl44* and *qgl3* leads to a genetic interaction that influences chalky grain ratio, protein content, gel consistency, and amylose content. *gnl44* and *gs3* had significant genetic interactions for chalky grain ratio, chalkiness degree, protein content, and amylose content. Conversely, *gs3* and *qgl3* exhibited genetic interactions for milled grain length, chalky grain ratio, and chalkiness degree (Table 2 and Table S3).

## 3. Discussion

### 3.1. The gnl44 Mutant Has Fewer but Larger Grains

In this study, we identified the mutant *gnl44* with a larger grain size from an EMS mutant library of the *japonica* rice cultivar Wuyunjing. Compared to WT, the *gnl44* mutant had a 20.2% increase in grain width and a 9.8% increase in grain length (Figure 1E,F). However, due to a concomitant drop in grain number per panicle, the final grain yield per plant decreased significantly compared to WT (Figure 1N). Using the MutMap approach for gene mapping, together with complementation experiments, we established that a G-to-A mutation in exon 7 of *Os02g0244100* led to the early termination of translation and was responsible for the mutant phenotype (Figure 3 and Figure 4). Subcellular localization revealed that GNL44 localized to the cytoplasm (Figure 5B). The results of tissue model expression showed that *gnl44* was highly expressed in the panicle and developing endosperm (Figure 5A). The Rice Annotation Project (rapdb.dna.affrc.go.jp, accessed on 6 May 2021) notes that *GNL44* encodes the RING-type E3 ubiquitin ligase GW2; the *gnl44* mutant therefore defines a new allele of *GW2*.

Lu et al. (2013) analyzed the *GW2* sequence across 127 rice varieties and reported that most SNPs/InDels were located in the intronic and promoter regions. Moreover, the variation in the promoter region did not affect *GW2* expression [37]. Zhang et al. (2015) and Dixit et al. (2013) analyzed *GW2* haplotypes in different rice resources and obtained similar results [36,38]. In this study, using EMS mutagenesis, we obtained a new mutant allele of *GW2* named *gnl44*. The *gnl44* mutant exhibits a severe reduction in grain number but also a larger grain phenotype, making *gnl44* a rare new allele of *GW2*. Song et al. (2007) cloned rice *GW2* using the cultivars WY3 and FAZ1. The authors determined that grain length, grain width, grain thickness, and thousand-grain weight were higher in NIL-*gw2* compared to FAZ1. However, grain number per panicle decreased and grain yield per plant increased in NIL-*gw2* [7]. Huang et al. (2022) independently cloned *GW2* using the cultivar HZ and observed that NIL-*gw2* had larger grains and a higher thousand-grain weight compared to HZ. However, there was no significant change in the grain number per panicle of NIL-*gw2*, and the yield per plant increased [39]. In the current study, we isolated the new *GW2* EMS mutant *gnl44*. Although *gnl44* grains were wider, longer, and heavier compared to WT grains, the number of grains per panicle decreased by 43.95% in *gnl44* compared to WT, resulting in a 21.8% decrease in grain yield per plant. Among the lines examined in these three studies, the phenotype of the NIL generated by Song et al. resulted from premature termination due to mutations in exon 4 of *GW2*, that of *gnl44* isolated in the current study resulted from premature termination due to mutations in exon 7, and that of the NIL isolated by Huang et al. resulted from a single base substitution in this gene. These findings suggest that exons 7 and 8 of *GW2* might be related to the establishment of grain number per panicle in rice.

GO enrichment analysis of the transcriptome data from young panicles at different developmental stages indicated that protein folding and other protein-related pathways were significantly enriched among the DEGs (Figure 4H,I). This finding is consistent with the observation that GNL44, an E3 ubiquitin ligase, is involved in protein degradation. Hao et al. (2021) found that GNL44/GW2 ubiquitinates the glutaredoxin protein OsGRX8 and targets it for degradation to regulate grain size. The modified expression of *OsGRX8* affects plant responses to various abiotic stresses [40]. Indeed, we found that many abiotic stress-related pathways were enriched in the transcriptome results (Figure 4H,I and Figure 7G,H). These results suggest that GNL44/GW2 may be involved in abiotic stress responses.

### 3.2. gnl44 Affects Appearance Quality and ECQ

*GW2* is a major gene affecting grain size, and previous research has mostly focused on its regulation of grain size [7,39,41,42]. Few studies have explored the regulation of rice quality by *GW2* in depth. In this study, we investigated the appearance and cooking qualities of the *gnl44* mutant. Compared to WT, chalky grain ratio and chalkiness percentage of the *gnl44* mutant increased (Figure 6G,H). The starch in *gnl44* mutant grains was loosely arranged (Figure 6E,F). Moreover, the observed distribution of amylopectin chain length suggests that *GNL44* mainly affected the starch biosynthesis of fa and fb1 chains (Figure 7D). The appearance quality of milled *gnl44* grain was worse than that of WT. In addition, the amylose and protein contents of *gnl44* grains was significantly decreased compared to WT (Figure 7A). The results of RVA showed that the peak viscosity and breakdown viscosity of *gnl44* were also decreased (Figure 7D,E). The taste value of the *gnl44* mutant was lower than that of WT (Figure 7F). These results indicate that *GNL44* mutation leads to poor appearance and taste quality in milled rice.

Song et al. (2007) [7] previously showed that amylose content, protein content, and gel consistency did not differ between NIL-*gw2* and FAZ1. However, the chalky grain ratio of NIL-*gw2* was increased compared to FAZ1. Based on these results, Song et al. [7] postulated that the *GW2* allele from cultivar WY3 had little influence on appearance and would not reduce cooking quality. Huang et al. (2022) [39] showed that amylose content, chalkiness degree, and chalky grain ratio similarly did not differ between NIL-*gw2* and HZ. However, the gel consistency of NIL-*gw2* was higher than that of HZ, while its protein content was lower than that of HZ. This observation was taken to indicate that the jf42-*GW2* allele did not affect the appearance quality of milled rice.

*Wx* is the major QTL affecting the appearance quality, eating quality, and cooking quality of rice [43,44]. To date, at least eight *Wx* alleles have been functionally characterized, including *Wx^lv^*, *Wx^a^*, *Wx^in^*, *Wx^b^*, *Wx^op^*^/*hp*^, *Wx^mq^*, *Wx^mp^*, and *wx* [44,45,46,47,48]. In this study, the *Wx* genotype of WYJ, *gnl44*, and TD70 was *Wx^b^*, and the *Wx* genotype of Kasalath, the monogenic NILs, and the two-gene NILs was *Wx^lv^*. We determined that in the *Wx^b^* background, the AC content decreased in the presence of the *GNL44*/*GW2* mutation (Figure 7A), while the same mutation increased the AC content in the *Wx^lv^* background (Figure 8F). These results indicate that although *GNL44*/*GW2* can affect AC, *Wx* is still the major gene determining AC.

Rice grain length and grain width are important factors affecting appearance quality. We investigated the grain length-to-width ratio in all three *GW2* studies. The grain length-to-width ratio was reported to be about 3.3 (Song et al., 2007 [7]), 2.91 (Huang et al., 2022 [39]), or 1.98 (this work). Therefore, the grain length-to-width ratio of *gnl44* in this study was significantly lower than that of *gw2* alleles reported in the other two studies, offering a possible explanation for the deterioration in appearance quality and cooking quality of *gnl44*.

Achary et al. [49] used clustered regularly interspaced short palindromic repeat (CRISPR)/CRISPR-associated nuclease 9 (Cas9)-mediated gene editing to target the fourth exon of *GW2*. The endosperm of the *GW2*-KO mutant seeds showed a thicker aleurone layer, with higher protein content in the seeds. The accumulation of essential dietary minerals (Fe, Zn, K, P, Ca) in the rice endosperm of the *OsGW2*-*KO* mutant also increased [49]. This result indicates that *GW2* not only affects the appearance and cooking quality but also the mineral content of rice.

The excessive accumulation of ROS in rice endosperm can trigger programmed cell death (PCD), thereby inhibiting the accumulation of storage substances and leading to chalkiness. The imbalance of redox homeostasis caused by oxidative stress is the main cause of grain chalkiness [50,51]. In this study, analysis of transcriptome data revealed the significant enrichment of genes involved in the oxidative stress response and protein metabolism-related pathways in *gnl44* grains (Figure 7G,H). These results suggest that *GNL44* may regulate grain appearance and ECQ by affecting the metabolism of rice quality-related proteins and redox homeostasis.

### 3.3. Potential Value of gnl44 for Rice Quality Improvement

In rice breeding, it is necessary to select the appropriate grain size gene to achieve a desired improvement in yield and quality [35]. Haplotype analysis indicates that the functional variation in *GW2* has largely not been exploited by breeders [37,38,52]. Therefore, evaluating the effects of different grain size genes, especially *gw2* and others, is of great significance for rice breeding in general and for rice quality in particular.

In this study, we isolated a rare new allele of *gw2*—*gnl44*. To assess the potential breeding value of *gnl44*, we used monogenic NILs and two-gene NILs carrying *gnl44*, *gs3*, or *qgl3* alleles to study their additive effects on traits related to rice quality. The amylose content of all three two-gene NILs decreased compared to that of Kasalath (Figure 9F). The amylose and protein contents of NIL-*gnl44*/*qgl3* were lower than those of Kasalath, but its gel consistency was higher than that of Kasalath (Figure 9F–H). Milled rice is reported to have a low amylose content, low protein content, and high gel consistency with good cooking quality [53]. We hypothesize that pyramiding of *gnl44* and *qgl3* can be a useful approach to improving the cooking quality of rice. However, the grain yield per plant of NIL-*gnl44*/*qgl3* was lower than that of Kasalath (Table S4), making it unsuitable for breeding rice varieties with high yield and good quality. The chalkiness degree, chalky grain ratio, and gel consistency of NIL-*gs2*/*qgl3* were lower than those of Kasalath, while its amylose and protein contents were higher than those of Kasalath (Figure 9D–H). Moreover, the grain yield per plant of NIL-*gs2*, *qgl3* was comparable to that of Kasalath (Table S4). Therefore, we believe that these two genes can be applied to the breeding of new rice varieties with high yield and good quality. The chalkiness grain rate and chalkiness degree of NIL-*gnl44*/*gs3* were much higher than those of Kasalath, and the highest among the three two-gene NILs were characterized here (Figure 9D,E). The appearance quality of NIL-*gnl44*, *gs3* was poor. Compared to Kasalath, NIL-*gnl44*/*gs3* had a lower amylose content, lower gel consistency, and higher protein content (Figure 9F–H). The grain yield per plant of NIL-*gnl44* and *gs3* was also lower than that of Kasalath (Table S4). Therefore, *gnl44* and *gs3* are not suitable for breeding new rice varieties with high yield and good quality.

## 4. Materials and Methods

### 4.1. Plant Materials and Growth Conditions

The *gnl44* mutant was obtained by mutagenizing the *japonica* rice (*Oryza sativa*) cultivar Wuyunjing (WYJ) with 0.8% ethyl methane sulfonate (EMS). We selected the *gnl44* mutant from the M2 generation. After 5 years of cultivation, its phenotype is stable and was used in this study. All plants in this study were cultivated at the Jiangsu Academy of Agricultural Sciences located in Nanjing, Jiangsu province (118°5′ E, 32°0′ N). The following agronomic traits were determined for field-grown plants at maturity: plant height, panicle length, grain number per panicle, primary and secondary branches per panicle, grain width, grain length, grain thickness, grain yield per plant, and thousand-grain weight [7].

### 4.2. Histological Analysis

Young spikelet hulls of wild-type (WT) and *gnl44* plants were fixed in FAA (50% ethanol, 5% glacial acetic acid, and 5% formaldehyde, all *v*/*v*) for 48 h. The samples were treated as described by Li et al. [54]. The stained sections were observed with a microscope (Olympus BX51, Tokyo, Japan). For glume cell observation, glumes of WT and *gnl44* were fixed in 2.5% (*w*/*v*) glutaraldehyde (2.5% [*w*/*v*] glutaraldehyde, 19.5% 2 M NaH_2_PO_4_, and 30.5% 2 M Na_2_HPO_4_) for 48 h and observed by scanning electron microscopy (Zeiss, EVO-LS10, Oberkochen, Germany).

### 4.3. Positional Cloning of GNL44

An F_2_ population was produced by crossing *gnl44* with the WT cultivar. WYJ was used to clone *GNL44*. The whole genomes of a mixed pool of 50 plants with the *gnl44* phenotype and wild-type WYJ were sequenced using a NextSeq 500 system (Illumina, San Diego, CA, USA). The MutMap was used to isolate *GNL44* as previously described [55]. The reference rice genome IRGSP1.0 was used in this study.

For complementation of the *gnl44* mutant, a 8330 bp genomic fragment of the candidate *GNL44* gene, which contains the entire genomic coding region of *GNL44*; 1662 bp of upstream sequence; and 787 bp of downstream sequence were amplified with the GNL44-COMF/R primers and sequenced (Table S1). The amplified fragment was cloned into the *BamH*I site of the pCAMBIA1300 binary vector. The resulting pCAMBIA1300-GNL44 plasmid was introduced into Agrobacterium (*Agrobacterium tumefaciens*) strain EHA105. *gnl44* calli were produced by the induction of mature embryos according to the descriptions of Toki (1997) and Toki (2006) [56,57]. Then, *gnl44* calli were transformed by an Agrobacterium-mediated transformation method.

### 4.4. Subcellular Localization of GNL44

The full-length *GNL44* coding sequence without the stop codon was amplified and cloned in-frame and upstream of the *GFP* coding sequence into the *Sal* I site of the p*35S::GFP* vector. The inserted fragment was amplified by PCR using the specific primer pairs shown in Table S1. The resulting construct was transfected into protoplasts isolated from 20-day-old 93-11 rice seedlings, which were incubated overnight in the dark as described previously [58]. GFP fluorescence was visualized with a confocal laser scanning microscope (Zeiss LSM 710).

### 4.5. RT-qPCR and RNA-seq Analysis

Total RNA was extracted from rice tissues using a Total RNA Miniprep kit (Axygene, Hangzhou, China) for RT-qPCR. First-strand cDNA was synthesized from total RNA using a ReverTra Ace qPCR-RT kit (Toyobo, Osaka, Japan), according to the manufacturer’s instructions. qPCR was performed using SYBR premix Ex Taq II (Takara, Kusatsu, Japan) on an Applied Biosystems 7900HT instrument. The primers used for qPCR are listed in Table S1. Rice *UBQ5* was used as an internal control. Data were analyzed following the relative quantification method [59]. Values are means ± SD of three biological replicates. Student’s *t*-test was used for statistical analysis.

For RNA-seq analysis, RNA samples were prepared from 3 cm (P1) and 10 cm (P2) panicles and the seeds of WT and *gnl44* plants at 5 and 10 days after flowering (DAF). RNA-seq analysis and Gene Ontology (GO) enrichment analysis were performed as described previously [60].

### 4.6. Determination of Taste Value and Rapid Visco Analyzer (RVA) Profiles

An RCTA-11A Taste Analyzer (Satake, Hiroshima, Japan) was used to analyze the taste value of milled rice according to the description of Chen et al. [61]. A 30 g aliquot of washed milled rice was transferred into a 50 mL aluminum box containing 40 mL distilled water and cooked in an electric rice cooker. After 20 min at equilibrium, the taste value of cooked rice was evaluated.

The RVA profiles of rice flour were investigated with a Rapid Visco Analyzer (Techmaster, Newport Scientific; Warriewood, Sydney, Australia). A 2.0 g aliquot of rice flour and 25 mL distilled water were mixed well in the RVA aluminum canister. The sample was heated according to the method described by Li et al. [62]. Finally, peak viscosity, cold viscosity, final viscosity, breakdown viscosity, setback viscosity, and pasting temperature were measured.

### 4.7. Measurement of Physicochemical Characteristics

The amylose content of rice flour was determined as described by Shi et al. [63]. The protein content was estimated from the nitrogen content using the Kjeldahl method with a conversion factor of 5.95 [64]. The gel consistency was measured following the method of Tan et al. [65].

### 4.8. Amylopectin Branch Chain Length Distribution Analysis

A high-performance anion-exchange chromatography system (ICS-6000, Thermo Fisher Scientific, Waltham, MA, USA) coupled with a Dionex™ CarboPac™ PA10 anion-exchange column was used following the method of Li et al. [62] with modifications to analyze the length distribution of amylopectin chains.

Starch samples (5 mg) were suspended in 1 mL double-distilled water and boiled for 30 min. Gelatinized polyglucan (500 μL) was mixed with 10 μL 1 m sodium acetate buffer, 2 μL sodium azide (2% solution, *w*/*v*), and 5 μL isoamylase (1000 U/μL 15284, Sigma Aldrich Corporation, St. Louis, MO, USA) and stored in a thermotank at 40 °C for 24 h. Samples were transferred to a boiling water bath for 10 min to terminate the reaction. After ultra-filtration, 500 μL of the solution was taken for analysis of the distribution of amylopectin branch chain length. The branching degree of starch was measured using a Bruker BioSpin GmbH nuclear magnetic resonance spectrometer (Bruker, Rheinstetten, Germany) following the method of Zou et al. [66].

### 4.9. Construction of NILs

All NILs in this study used Kasalath as the recipient material. For NIL-*gnl44*, the *gnl44* mutant was used as a donor; for NIL-*gs3* and NIL-*qgl3*, TD70 was used as donor. After five generations of repeated backcrossing, the BC_5_F_1_ population was obtained, from which the BC_5_F_3_ population was obtained after two generations of selfing.

From the BC_5_F_3_ generation, we developed near-isogenic lines for *qgl3*(NIL-*qgl3*) and *gs3*((NIL-*gs3*)), which carry an ∼103.5- and ∼118.4-kb segment of TD70 containing the *qgl3* and *gs3* loci in the Kasalath genetic background, respectively, using molecular marker-assisted selection (MAS). NIL-gnl44 was constructed with a very small *gnl44* chromosomal region (~123.6 kb) containing the *gnl44* locus in the Kasalath genetic background using MAS. An analysis of the genes in these segments revealed that they do not contain known grain size- or quality-related genes.

For the two-gene NILs, NIL-*gnl44*, NIL-*gs3*, and NIL-*qgl3* were crossed to obtain F_1_ seeds. In 2018, F_1_ plants were grown and F_2_ seeds were harvested in Nanjing, Jiangsu Province, China. F_2_ plants were planted in Hainan Province, China, in 2018, and plants homozygous for *gnl44* and *gs3* (NIL-*gnl44*/*gs3*) or *gnl44* and *qgl3* (NIL-*gnl44*/*qgl3*) were selected from the F_2_ family using MAS.

## 5. Conclusions

In this study, we functionally characterized the effects of *GNL44* on grain size, appearance quality, and ECQ in rice. The *gnl44* mutant had fewer but larger grains than the WT. The appearance quality and taste value of the *gnl44* mutant were reduced. The phenotype of *gnl44* is caused by a mutation in the *Os02g0244100* gene. We also explored the effects of *gnl44*, *gs3*, and *qgl3* on rice quality using monogenic NILs and two-gene NILs. Analysis of the monogenic NILs showed that *gnl44* and *qgl3* mainly affected chalkiness and gel consistency, and *gs3* mainly affected protein content. Analysis of the grain quality and ANOVA of the two-gene NILs showed that *gnl44* and *gs3* had significant genetic interactions for chalky grain ratio, chalkiness degree, protein content, and amylose content. The genetic interaction of *gnl44* and *qgl3* influences chalky grain ratio, protein content, gel consistency, and amylose content. The genetic interaction of *gs3* and *qgl3* affects milled grain length, chalky grain ratio, and chalkiness degree. Our results lay the foundation for breeding rice cultivars with high yields and superior quality.

## Figures and Tables

**Table 1:** Genetic analysis of *gnl44.*

Crossing Combination	Phenotype of F_1_ Plants	Number of F_2_ Plants		χ^2^ (3:1 = 3.84)
		Normal Grains	Large Grains	
*gnl44* × Wuyunjing	Normal grains	487	119	2.5782
Wuyunjing × *gnl44*	Normal grains	503	122	2.7002

**Table 2:** Two-way analysis of variance for genetic interactions among *gnl44*, *qgl3*, and *gs3.*

Trait	Variation	*df*	SS	MS	F	*p*
Milled grain length	*gs3*	1	4.66	4.66	24.33	<0.0001
	*qgl3*	1	8.96	8.96	46.8	<0.0001
	*gs3* × *qgl3*	1	1.13	1.13	5.92	0.0201
	error	36	6.89	0.19		
Chalky grain ratio	*gnl44*	1	4428	4428	417.08	<0.0001
	*gs3*	1	1245	1245	117.29	<0.0001
	*gnl44* × *gs3*	1	1118	1118	105.27	<0.0001
	error	16	169.9	10.62		
	*gnl44*	1	2488.2	2488.2	510.55	<0.0001
	*qgl3*	1	38.4	38.4	7.87	0.0127
	*gnl44* × *qgl3*	1	281.25	281.25	57.71	<0.0001
	error	16	78.0	4.87		
	*gs3*	1	446.0	446.0	34.61	<0.0001
	*qgl3*	1	13.7	13.7	1.06	0.318
	*gs3* × *qgl3*	1	371.1	371.1	28.79	<0.0001
	error	16	206.2	12.9		
Chalkiness degree	*gnl44*	1	499.6	499.6	2435.97	<0.0001
	*gs3*	1	54.65	54.65	266.46	<0.0001
	*gnl44* × *gs3*	1	60.4	60.41	294.56	<0.0001
	error	16	3.3	0.20		
	*gs3*	1	8.3	8.3	25.74	0.0001
	*qgl3*	1	15.8	15.8	49.16	<0.0001
	*gs3* × *qgl3*	1	10.6	10.6	32.98	<0.0001
	error	16	5.2	0.32		
Protein content	*gnl44*	1	0.89	0.89	18.34	0.0027
	*gs3*	1	0.10	0.10	2.09	0.1864
	*gnl44* × *gs3*	1	0.49	0.49	10.11	0.013
	error	8	0.39	0.048		
	*gnl44*	1	0.235	0.235	9.18	0.0163
	*qgl3*	1	0.006	0.006	0.22	0.6517
	*gnl44* × *qgl3*	1	0.529	0.529	20.64	0.0019
	error	8	0.205	0.026		
Gel consistency	*gnl44*	1	121.0	121.0	10.62	0.0068
	*qgl3*	1	81.0	81.0	7.11	0.0205
	*gnl44* × *qgl3*	1	728.7	728.7	63.99	<0.0001
	error	12	136.7	11.39		
Amylose content	*gnl44*	1	2.47	2.47	5	0.0557
	*gs3*	1	3.56	3.56	7.23	0.0276
	*gnl44* × *gs3*	1	8.74	8.74	17.72	0.003
	error	8	3.945	0.49		
	*gnl44*	1	0.12	0.12	0.25	0.6284
	*qgl3*	1	5.87	5.87	12.59	0.0075
	*gnl44* × *qgl3*	1	2.99	2.99	6.42	0.0351
	error	8	3.73	0.47		

Df: degree of freedom; SS: sum of squares; MS: mean of square; F: F value of ANOVA.

## Data Availability

The data presented in this study are available upon request from the corresponding author.

## Supplementary Materials

The following supporting information can be downloaded at: https://www.mdpi.com/article/10.3390/ijms25010589/s1.
