# Supplementary material for: The RING-Type Domain-Containing Protein GNL44 Is Essential for Grain Size and Quality in Rice (Oryza sativa L.)
Source: Int J Mol Sci. 2024 Jan 2;25(1):589. doi: 10.3390/ijms25010589 (PMC10779214; doi:10.3390/ijms25010589)
Supplement: Supplementary file 1 [file ijms-25-00589-s001.zip › ijms-2759637-supplementary.pdf]

Table S1. Primers used in this study.

Primer	Sequence (5’ to 3’)	Use
GNL44-COMF	gctcggtacccggggatccGGGTGTGTTTAGTTCACGCC	complementation
GNL44-COMR	aggtcgactctagaggatccAGCCTCGAGGTTATGGAATCG	complementation
real GNL44F	GCGATTTGGCGTTCCATTCA	RT-qPCR
real GNL44R	AGAGAAGCAGCTGGAACGAA	RT-qPCR
GNL44GFPF	tatttacaattacagtcgacATGGGGAACAGGATAGGGGG	GFP
GNL44GFPR	atggatcctctagagtcgacCAACCATGCCAACCCTTGCG	GFP

Table S2. Rapid Visco Analyzer profile characteristics of WT and *gnl44*.

lines	Peak viscosity (cP)	Cold viscosity (cP)	Breakdown viscosity (cP)	Final viscosity (cP)	Setback viscosity (cP)	Consistency viscosity (cP)	Peak Time (min)	Pasting temperature (°C)
WT	3487±39.11	2140.67±16.95	1346.33±22.39	3018±2.08	-469±37.03	877.33±14.88	6.3±0.1	73.63±0.35
gnl44	3300.33±25.08*	2266.33±4.91**	1034±29.37**	3096.33±16.19**	-204±40.46**	830±11.36	6.32±0.078	72.73±0.43

Values are Mean±SD (n = 3). Student's t-test was used to generate P values; * $P < 0.05$ and ** $P < 0.01$.

Table S3. Two-way analysis of variance for genetic interactions among *gnl44*, *gs3*, and *qgl3.*

Trait	Variation	df	SS	MS	*F*	*P*
Milled grain length	*gnl44*	1	1.406	1.406	2.97	0.0936
	gs3	1	4.802	4.802	10.13	0.003
	gnl44×gs3	1	1.204	1.204	2.54	0.1197
	error	36	17.066	0.474		
	gnl44	1	1.739	1.739	5.15	0.0294
	qgl3	1	8.372	8.372	24.79	<0.0001
	gnl44×qgl3	1	0.930	0.930	2.75	0.1057
	error	36	12.160	0.338		
Milled grain width	*gnl44*	1	0.593	0.593	3.06	0.0888
	gs3	1	0.042	0.042	0.21	0.6459
	gnl44×gs3	1	0.013	0.013	0.07	0.8002
	error	36	6.976	0.194		
	gnl44	1	0.617	0.617	5.08	0.0304
	qgl3	1	0.035	0.035	0.29	0.5929
	gnl44×qgl3	1	0.016	0.016	0.13	0.7156
	error	36	4.379	0.122		
	gs3	1	0.253	0.253	1.55	0.2215
	qgl3	1	0.222	0.222	1.36	0.2513
	gs3×qgl3	1	0.169	0.169	1.03	0.3158
	error	36	5.879	0.163		
Chalkiness degree	*gnl44*	1	212.813	212.813	696.42	<0.0001
	qgl3	1	52.294	52.294	171.13	<0.0001
	gnl44×qgl3	1	0.0001	0.0001	0	0.9873
	error	16	4.889	0.306		
Protein content	*gs3*	1	0.902	0.902	19.22	0.0023
	qgl3	1	0.755	0.755	16.09	0.0039
	gs3×qgl3	1	0.004	0.004	0.09	0.7671
	error	8	0.375	0.047		
Gel consistency	*gnl44*	1	358.06	358.06	38.3	<0.0001
	gs3	1	291.64	291.64	31.19	0.0001
	gnl44×gs3	1	8.57	8.57	0.92	0.3573
	error	12	112.20	9.35		
	gs3	1	119.57	119.57	15.87	0.0018
	qgl3	1	218.45	218.45	29	0.0002
	gs3×qgl3	1	10.34	10.34	1.37	0.2642
	error	12	90.41	7.53		
Amylose content	*gs3*	1	0.005	0.005	0.02	0.8983
	qgl3	1	3.36	3.36	11.24	0.0101
	gs3×qgl3	1	1.30	1.30	4.35	0.0706
	error	8	2.39	0.300		
Grain number per panicle	*gnl44*	1	24790.7	24790.7	54.18	<0.0001
	gs3	1	3896.5	3896.5	8.52	0.0101

gnl44×gs3	1	11360.2	11360.2	24.83	0.0001
error	16	7320.3	457.5		
gnl44	1	113537.4	113537.4	238.24	<0.0001
qgl3	1	12958.1	12958.1	27.19	<0.0001
gnl44×qgl3	1	5317.1	5317.1	11.16	0.0042
error	16	7625.05	476.6		
gs3	1	14571.9	14571.9	28.32	<0.0001
qgl3	1	13798.6	13798.6	26.82	<0.0001
gs3×qgl3	1	5860.2	5860.2	11.39	0.0039
error	16	8232.6	514.5		

df, degree of freedom; SS, sum of squares; MS, mean of square; *F, F* value of ANOVA.

Table S4. Agronomic traits of Kasalath and various near-isogenic lines (NILs).

lines	Number of panicles per plant	Panicle length (cm)	Number of primary branches per plant	Number of secondary branches per plant	Seed-setting rate (%)	Grain yield per plant (g)
Kasalath	11.0 ± 0.0 a	27.0 ± 0.6 bc	9.9 ± 0.4 ab	41.6 ± 1.1 a	89.3 ± 5.3 a	21.0 ± 0.6 ab
NIL-*gnl44*	10.7 ± 1.15 a	25.5 ± 1.3 c	9.1 ± 1.0 bc	41.2 ± 5.6 a	89.3 ± 2.9 a	22.5 ± 3.5 a
NIL-*qgl3*	11.3 ± 1.5 a	30.7 ± 0.8 a	10.3 ± 0.3 a	42.9 ± 2.9 a	85.0 ± 2.8 a	24.0 ± 2.4 a
NIL-*gs3*	10.0 ± 0.0 ab	25.4 ± 0.8 c	8.9 ± 0.1 c	38.7 ± 2.4 ab	75.1 ± 3.7 b	14.6 ± 0.35 c
NIL-*gnl44/gs3*	10.3 ± 1.45 a	22.6 ± 0.65 d	8.5 ± 0.2 c	31.1 ± 1.8 cd	87.2 ± 5.9 a	17.1 ± 1.4 bc
NIL-*gnl44/qgl3*	8.4 ± 0.7 b	28.6 ± 0.8 b	8.6 ± 0.4 c	29.3 ± 1.8 d	87.15 ± 1.5 a	15.6 ± 3.1 c

Values are means ± SD (n = 10). Different lowercase letters indicate significant differences as determined by ANOVA followed by Fisher's LSD test for the comparison of means (P < 0.05).
